# Letter to the Editor in response to “Epidemiological trends in enamel hypomineralization and molar-incisor hypomineralization: a systematic review and meta-analysis" Clinical oral investigations vol. 29,6 327. 2 Jun. 2025, doi:10.1007/s00784-025-06411-4

**DOI:** 10.1007/s00784-026-06802-1

**Published:** 2026-03-04

**Authors:** Azza Tagelsir Ahmed

**Affiliations:** Department of Pediatric Dentistry College of Dentistry, University of Illinois Chicago, Chicago, USA

Dear Editor,

I read with great interest the recently published article titled *“Epidemiological trends in enamel hypomineralization and molar-incisor hypomineralization: a systematic review and meta-analysis”* by Ammar, Nour et al., 2025, which addresses an important topic in clinical oral health research. The authors should be commended for their contribution, particularly their efforts to study a crucial oral public health issue (EH/MIH) and their accompanying trends across the different regions of the world.

However, I wish to draw attention to the omission of a key U.S. epidemiological study that meets the review’s inclusion criteria and provides nationally relevant data “*Tagelsir Ahmed A*,* Soto-Rojas AE*,* Dean JA*,* Eckert GJ*,* Martinez-Mier EA. Prevalence of molar-incisor hypomineralization and other enamel defects and associated sociodemographic determinants in Indiana. The Journal of the American Dental Association. 2020;151(7):491–501*”.

This study involved a reasonably large, representative American cohort and reported MIH prevalence and diagnostic criteria consistent with the methods applied by Ammar et al. Its inclusion could have strengthened the North American dataset, potentially altering the pooled estimates reported by the mentioned study. The study had a rigorous, standardized clinical examination protocol, conducted by a single calibrated examiner using both the Modified Developmental Defects of Enamel (DDE) Index and the European Academy of Pediatric Dentistry (EAPD) criteria. This approach ensured high diagnostic validity and produced high-quality, comparable epidemiological data—a feature not consistently present in many MIH prevalence studies worldwide. Systematic reviews of this scope provide foundational prevalence data; therefore, comprehensive coverage of available epidemiological studies is critical.

## Data Availability

No datasets were generated or analysed during the current study.

